# Assessing territorial disparities in snakebite surveillance data in Brazil: Implications for public health

**DOI:** 10.1371/journal.pntd.0013873

**Published:** 2026-01-16

**Authors:** Alexandre Vilhena Silva-Neto, Gabriel dos Santos Mouta, Thaís Pinto Nascimento, Antônio Alcirley da S. Balieiro, Jady Shayenne Mota Cordeiro, Jefferson Valente, André Sachett, Patrícia Carvalho da Silva Balieiro, Djane Clarys Baia-da-Silva, Fernando Almeida-Val, Wuelton Marcelo Monteiro, Vanderson de Souza Sampaio

**Affiliations:** 1 Universidade do Estado do Amazonas, Manaus, Brasil; 2 Fundação de Medicina Tropical Doutor Heitor Vieira Dourado, Manaus, Brasil; 3 Instituto Leônidas & Maria Deane, Fundação Oswaldo Cruz, Manaus, Amazonas, Brasil; 4 Fundação de Vigilância em Saúde do Amazonas, Manaus, Brasil; 5 Instituto Todos pela Saúde, São Paulo, Brazil; Universidad de Costa Rica, COSTA RICA

## Abstract

**Background:**

Snakebite envenoming (SBE) is a neglected tropical disease with high incidence and impact in rural and remote areas of Brazil. Although the country maintains a national reporting system (SINAN) for SBE, few studies have systematically evaluated the quality of these surveillance data, which are essential for informing public health planning and antivenom distribution strategies.

**Methodology:**

This is a descriptive study analyzing all snakebite notifications recorded in SINAN between 2007 and 2023. Data quality was evaluated across three dimensions: completeness, internal consistency, and reliability. Sociodemographic, clinical, and treatment-related variables were assessed by region using standardized classifications. Inter-database agreement for fatal outcomes was analyzed between SINAN and the Mortality Information System (SIM) using the Intraclass Correlation Coefficient (ICC).

**Findings:**

The results showed excellent completeness for core demographic variables such as sex and age, but poor or very poor completeness for education level, occupation, race/ethnicity, and clinical manifestations. Internal inconsistencies, although infrequent, were present across all regions. The overall ICC for fatal outcomes was 0.95, with regional variation. Antivenom use was frequently inconsistent with national treatment guidelines, particularly in cases involving *Crotalus*, *Lachesis*, and *Micrurus* genera.

**Conclusions and significance:**

Despite the availability of a national notification system, snakebite surveillance in Brazil faces substantial limitations in data completeness and consistency, especially for clinical and treatment variables. These gaps are more pronounced in high-burden and remote regions, such as the Amazon, and may hinder equitable resource allocation and policy development. Strengthening notification practices, investing in training, and integrating clinical and epidemiological workflows are critical to improving the quality and utility of SBE data for public health action.

## Author summary

Snakebite envenoming remains a significant but often overlooked public health issue in Brazil, particularly in rural and remote regions where Indigenous and underserved communities are most affected. Although Brazil has a national notification system (SINAN) to track cases and guide antivenom distribution, the quality of this data varies widely across regions. In this study, we analyzed over 500,000 snakebite records from 2007 to 2023 to evaluate the completeness, consistency, and reliability of surveillance data. We found that while basic demographic information is usually well reported, important clinical and treatment-related details are frequently missing or inconsistent—especially in regions with the highest burden of disease, such as the Amazon. In some cases, antivenom use did not align with national guidelines. Our findings suggest that strengthening surveillance practices and adapting reporting systems to the realities of remote and intercultural settings are critical to improving snakebite care and reducing health disparities in Brazil.

## Introduction

Snakebite envenoming (SBE) remains a serious yet underreported public health problem in many low- and middle-income countries, especially in tropical regions. Globally, SBEs are estimated to cause up to 138,000 deaths and 400,000 permanent disabilities every year, mostly in rural and underserved communities [1,2]. The World Health Organization (WHO) recognizes SBE as a Category A neglected tropical disease (NTD), emphasizing the need for improved surveillance and access to antivenom as part of global efforts to reduce the burden of neglected conditions [3,4].

In Brazil, snakebites are part of the list of notifiable health conditions and are monitored through the Notifiable Diseases Information System (Sistema de Informação de Agravos de Notificação – SINAN). This system plays a central role in organizing epidemiological data, specially supporting the antivenom distribution, and informing public health policies. Inconsistencies in filling out notification data potentially impact the distribution and planning of antivenom implementation programs and other infrastructure and human resources for the care of snakebite patients [5,6]. However, several studies have documented persistent limitations in the quality of notification data, including missing fields, internal inconsistencies, and poor reliability in key variables such as case severity, antivenom use, and time to care [6–8].

These limitations are not merely technical. Incomplete or inaccurate surveillance data can distort risk mapping, delay responses, and compromise the allocation of antivenom stocks — particularly in remote areas where access to care is already limited [9,10]. In the Brazilian Amazon, where nearly half of all SBE cases occur, challenges related to geography, health system infrastructure, and logistics further aggravate these problems [11].

The Indigenous Health Subsystem, responsible for delivering care to Indigenous populations, faces additional constraints. Although its professionals are on the frontline of snakebite management in the Amazon, they often operate without stable internet access, appropriate refrigeration for antivenom, or training in surveillance protocols [10,12]. As a result, notification quality tends to be especially fragile in districts with the highest burden of disease, undermining both clinical and strategic responses [8,7].

Despite the critical role of SINAN in national SBE surveillance, few studies have systematically evaluated the quality of its snakebite records using structured frameworks. Previous assessments have focused primarily on disease trends or antivenom effectiveness, rather than on the completeness and consistency of the data itself [13]. Yet understanding the quality of these records is essential for interpreting epidemiological patterns and improving health governance in vulnerable regions.

This study aims to fill this gap by evaluating the quality of snakebite notifications in Brazil from 2007 to 2023. We focus on three core dimensions of data quality: completeness, internal consistency, and reliability. By identifying systemic weaknesses in notification practices, we seek to support efforts to strengthen surveillance, improve antivenom management, and enhance the responsiveness of the public health system — particularly in regions most affected by snakebites.

## Methods

### Ethics statement

This study used secondary data that is anonymized and publicly accessible. According to Resolution No. 510/2016 of the Brazilian National Health Council, studies using such data are exempt from ethical review.

### Study design and data source

This is a descriptive study based on epidemiological surveillance data of snakebite envenoming (SBE) cases reported in Brazil through the Notifiable Diseases Information System (Sistema de Informação de Agravos de Notificação – SINAN) between January 1, 2007, and December 31, 2023. SINAN is the national platform responsible for recording and monitoring a wide range of notifiable health conditions, including accidents involving venomous animals. Snakebite envenoming has been included on SINAN’s list of mandatory reportable conditions since 2001, and cases are documented using a standardized notification form specific to ophidic accidents. This form captures sociodemographic information (e.g., sex, age, race/ethnicity, education), clinical features (e.g., bite site, local and systemic manifestations, severity, complications), and details related to treatment and outcomes (e.g., time to medical care, antivenom administration, type, dosage, and clinical evolution).

SINAN plays a strategic role in Brazil’s national response to snakebites. The data it collects are used by the Ministry of Health to estimate antivenom demand, guide stock distribution, and plan the allocation of resources—including infrastructure and trained personnel—across states and municipalities. Consequently, limitations in data completeness and consistency, particularly for clinical severity and treatment variables, may compromise supply chain decisions and hinder the implementation of targeted interventions in high-burden areas. The antivenom allocation and health planning are directly dependent on this system.

The dataset used in this study was obtained from the open-access repository of the Department of Informatics of the Unified Health System (DATASUS), publicly available at https://datasus.saude.gov.br/transferencia-de-arquivos/. As the database is fully de-identified and contains no personal or sensitive information, its use complies with Brazilian public data access regulations.

Initial data processing and preparation, including database cleaning and structuring, were performed using the R programming language within the RStudio IDE (version 4.2). Geospatial shapefiles and cartographic references were prepared in QGIS version 3.14 to enable spatial visualization of key indicators in subsequent analyses.

The dataset, dictionary, and codes used in the analyses are available at the following GitHub repository https://github.com/AlexandreNetoEng/datasnakebite.

### Completeness assessment

To assess data completeness, we calculated the proportion of filled fields across all records for each variable of interest. Fields left blank or marked as “ignored” were considered incomplete. The percentage of completeness was determined by dividing the number of valid responses by the total number of notifications. To classify the level of completeness, we applied the framework proposed by Romero and Cunha [14], which categorizes variable completeness as excellent (≥95%), good (90–94.9%), regular (80–89.9%), poor (50–79.9%), or very poor (<50%). This assessment was performed for key variables grouped into three main blocks: sociodemographic data (such as age, sex, race/color, place of residence), clinical manifestations and case classification (including severity, symptoms, and complications), and treatment-related information (such as antivenom administration and time to care).

### Reliability assessment

The reliability of notification records was evaluated by comparing the SINAN database with the Mortality Information System (SIM) for cases where “death” was reported as an outcome. A reliable notification was defined as one in which death was consistently recorded in both systems. To quantify this agreement, we used the Intraclass Correlation Coefficient (ICC) following the average random raters’ model (ICC2k), which accounts for variation between coders across independent databases. The interpretation of ICC scores was based on the classification proposed by Koo and Li (2016): poor (<0.50), moderate (0.50–0.75), good (0.75–0.90), and excellent (>0.90) [15].

### Internal consistency assessment

Internal consistency was analyzed by identifying logically incompatible values within individual notifications, using validation rules derived from the official Ministry of Health instruction manual for completing the SINAN notification form for envenomation. One criterion was the presence of inconsistent age formats, such as invalid combinations in the four-digit coding structure used by SINAN (where the first digit indicates units: 1 = hours, 2 = days, 3 = months, 4 = years). We also checked for contradictions between parent fields and dependent subfields. For example, when the main field for “local manifestations” was blank or ignored but subfields such as “pain” or “edema” were filled, the record was marked inconsistent. The same logic was applied to systemic manifestations, complications, and other hierarchical field groups. In addition, we evaluated spatial inconsistencies by verifying mismatches between municipality and state codes in the fields for place of occurrence, residence, and healthcare unit. These inconsistencies were flagged when the first two digits of the municipality code, which identify the state, did not correspond across fields.

### Evaluation of antivenom use

To evaluate the adequacy of antivenom use, we compared the type and dosage recorded in SINAN with Brazilian Ministry of Health recommendations, which specify the appropriate antivenom according to the identified snake genus and the severity of the case. We classified antivenom administration into three categories: (i) inappropriate use of non-recommended antivenom, (ii) mixed use of recommended and non-recommended antivenom, and (iii) inappropriate dosage relative to case severity. For dosage assessment, cases were categorized as receiving the correct dose, an underdose, or an overdose, based on national therapeutic guidelines for mild, moderate, or severe envenomation [16].

### Statistical analysis

All statistical analyses were conducted using the R programming language (version 4.2) within the RStudio integrated development environment. Descriptive statistics were used to characterize the dataset and to summarize the dimensions of data quality. For categorical variables, absolute frequencies and relative percentages were calculated. The analysis was stratified by epidemiological variables such as year of notification, federal unit (state), and macroregion to identify temporal and geographic trends.

To evaluate completeness, we computed the proportion of filled fields for each variable by dividing the number of valid (non-null and non-“ignored”) responses by the total number of notifications. The results were expressed as percentages and categorized using the Romero & Cunha classification scale [14], as previously described. Comparative assessments between variables and across regions were visualized using heatmaps and color-coded tables.

For reliability, we applied the Intraclass Correlation Coefficient (ICC) using the irr package in R. The ICC2k model was selected to account for variability among different raters (data entry agents) between databases. The “death” variable was used as the reference for inter-database comparison between SINAN and the Mortality Information System (SIM). Confidence intervals for ICC values were computed at 95%, and agreement levels were classified as poor to excellent, following the thresholds proposed by Koo and Li (2016) [15].

Internal consistency was assessed through logic-based conditional rules coded in R. These included detecting contradictory field entries, such as the presence of detailed symptoms under empty parent categories or inconsistent demographic information. Inconsistency rates were calculated as the proportion of cases presenting logical conflicts relative to the total number of valid records for each variable set.

We also examined antivenom usage patterns, applying filters based on the Brazilian Ministry of Health clinical guidelines [16]. These filters enabled the identification of inappropriate use (wrong type), polytherapy (simultaneous use of correct and incorrect types), and incorrect dosage (based on case severity). The antivenom analysis was conducted using dplyr and tidyr packages to structure and manipulate records, while proportions of underdosage, correct dosage, and overdosage were computed and stratified by region and year.

Spatial visualizations were generated using the sf, tmap, and ggplot2 packages to map the geographic distribution of completeness, inconsistency, and antivenom adequacy across Brazilian municipalities. All results were summarized with descriptive statistics; no inferential tests were conducted, as the objective of the study was to evaluate data quality attributes rather than test hypotheses or associations.

## Results

**Fig 1:**
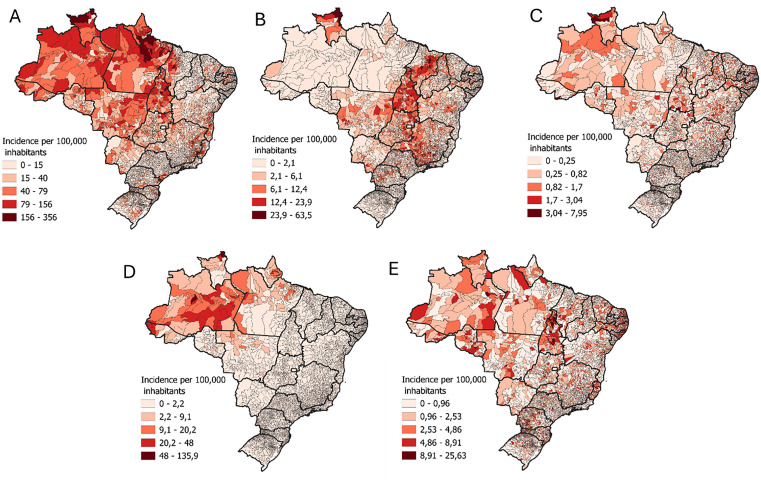
Geographic distribution of reported snakebite envenoming cases in Brazil (2007–2023), by municipality and trends in death reporting according to information systems (SINAM) across years. Spatial distribution of snakebite envenoming notifications across Brazilian municipalities based on SINAN data from 2007 to 2023. Higher case densities are observed in the North region, particularly in Amazonian states. Municipalities with no registered cases are shown in white. A – *Bothrops*; B – *Crotalus*; C – *Micrurus*; D – *Lachesis*; E - Non-venomous snakes. Source of shapefiles: Brazilian Institute of Geography and Statistics (IBGE), 2023. Shapefiles are available under the Creative Commons Attribution 4.0 License (CC BY 4.0): https://www.ibge.gov.br/geociencias/organizacao-do-territorio/malhas-territoriais/15774-malhas.html?=&t=acesso-ao-produto.

Between 2007 and 2023, a total of [503,737] cases of snakebite envenomation were reported to SINAN throughout Brazil. The distribution of cases by region showed the highest concentration in the North region [160,879], followed by the Northeast [136,386], Southeast [113,999] Central-West [50,344] and South [42,129] regions (S1 Fig). Most cases occurred in males (386,466/ 503,648 (76.7%), the mean age group was 32 ± 18 years. And in terms of race/skin color, [281,154/ 460,954 (60.9%)] of the cases were classified as mild 264,496/ 473,529 (55.8%) (S1 Table). The geographic distribution of cases among municipalities is shown in Fig 1.

The evaluation of data completeness revealed that core sociodemographic variables such as sex, age, and residence presented excellent completeness across most regions, with values above 98% (S2 Table). In contrast, race/skin color, education level, and occupation had poor or very poor completeness in several states. Clinical variables such as severity classification, location of bite, and case outcome generally showed good completeness. However, key variables such as clotting time, clinical manifestations, and complications had high rates of missing or ignored fields, especially in the North and Northeast. The field indicating whether antivenom was administered had excellent completeness, while fields describing the type, dose, and treatment regimen showed low completeness in several regions (Table 1).

**Table 1:** Completeness of selected variables in snakebite envenoming notifications in Brazil, by region (2007–2023). Completeness percentages for key sociodemographic, clinical, and treatment-related variables reported in SINAN, stratified by Brazilian geographic region. Classification of completeness levels follows Romero and Cunha’s criteria: excellent (≥95%), good (90–94.9%), regular (80–89.9%), poor (50–79.9%), and very poor (<50%).

Characteristic	Overall	Norte	Nordeste	Sudeste	Sul	Centro-Oeste
	N = 503,737^*1*^	N = 160,879^*1*^	N = 136,386^*1*^	N = 113,999^*1*^	N = 42,129^*1*^	N = 50,344^*1*^
**Year**						
2007	26,560 (5.3%)	7,902 (4.9%)	6,796 (4.9%)	6,564 (5.7%)	3,029 (7.1%)	2,269 (4.5%)
2008	27,787 (5.5%)	8,341 (5.1%)	6,934 (5.0%)	6,952 (6.1%)	2,764 (6.5%)	2,796 (5.5%)
2009	29,765 (5.9%)	9,148 (5.6%)	8,359 (6.1%)	6,315 (5.5%)	2,999 (7.1%)	2,944 (5.8%)
2010	29,657 (5.9%)	9,227 (5.7%)	8,183 (6.0%)	6,388 (5.6%)	2,717 (6.4%)	3,142 (6.2%)
2011	30,142 (5.9%)	9,096 (5.6%)	7,945 (5.8%)	7,301 (6.4%)	2,596 (6.1%)	3,204 (6.3%)
2012	28,336 (5.6%)	8,919 (5.5%)	6,808 (4.9%)	7,102 (6.2%)	2,422 (5.7%)	3,085 (6.1%)
2013	27,390 (5.4%)	9,521 (5.9%)	6,118 (4.4%)	6,785 (5.9%)	2,251 (5.3%)	2,715 (5.3%)
2014	26,209 (5.2%)	9,469 (5.8%)	5,889 (4.3%)	5,746 (5.0%)	2,352 (5.5%)	2,753 (5.4%)
2015	27,167 (5.3%)	9,062 (5.6%)	7,020 (5.1%)	5,766 (5.0%)	2,409 (5.7%)	2,910 (5.7%)
2016	26,589 (5.3%)	8,783 (5.4%)	7,103 (5.2%)	5,372 (4.7%)	2,392 (5.6%)	2,939 (5.8%)
2017	28,738 (5.7%)	8,898 (5.5%)	7,261 (5.3%)	6,903 (6.0%)	2,542 (6.0%)	3,134 (6.2%)
2018	30,476 (6.0%)	9,798 (6.0%)	7,904 (5.8%)	7,556 (6.6%)	2,396 (5.6%)	2,822 (5.6%)
2019	34,192 (6.7%)	11,102 (6.9%)	9,867/ (7.2%)	7,476 (6.5%)	2,452 (5.8%)	3,295 (6.5%)
2020	33,838 (6.7%)	10,709 (6.6%)	10,194 (7.4%)	7,488 (6.5%)	2,267 (5.3%)	3,180 (6.3%)
2021	33,157 (6.5%)	10,689 (6.6%)	10,265 (7.5%)	6,882 (6.0%)	2,137 (5.0%)	3,184 (6.3%)
2022	31,136 (6.1%)	10,423 (6.4%)	9,478 (6.9%)	6,459 (5.6%)	2,017 (4.7%)	2,759 (5.4%)
2023	32,598 (6.4%)	9,792 (6.0%)	10,262 (7.5%)	6,944 (6.0%)	2,387 (5.6%)	3,213 (6.3%)
**Age**	35, 19	32, 18	35, 19	39, 19	39, 19	37, 19
**Biological sex**						
Male	386,466/ 503,648 (76.7%)	126,611/ 160,864 (78.7%)	102,535/136,353 (75.2%)	87,030/ 113,973 (76.3%)	31,851/ 42,126 (75.6%)	38,439/ 50,332 (76.3%)
Female	117,182/ 503,648 (23.2%)	34,253/ 160,864 (21.2%)	33,818/ 136,353 (24.8%)	26,943/ 113,973 (23.6%)	10,275/ 42,126 (24.3%)	11,893/ 50,332 (23.6%)
Unknown	89	15	33	26	3	12
**Pregnancy data**						
1st Quarter	605/ 490377 (0.1%)	222/ 158311 (0.1%)	144/ 130997 (0.1%)	104/ 110538 (0.0%)	77/ 41507 (0.1%)	58/ 49024 (0.1%)
2nd Quarter	944/ 490377 (0.1%)	347/ 158311 (0.2%)	262/ 130997 (0.2%)	164/ 110538 (0.1%)	75/ 41507 (0.1%)	96/ 49024 (0.2%)
3nd Quarter	543/ 490377 (0.1%)	222/ 158311 (0.1%)	149/ 130997 (0.1%)	88/ 110538 (0.0%)	28/ 41507 (0.0%)	56/ 49024 (0.1%)
Gestational Age Unknown	517/ 490377 (0.1%)	214/ 158311 (0.1%)	186/ 130997 (0.1%)	65/ 110538 (0.0%)	14/ 41507 (0.0%)	38/ 49024 (0.0%)
Not pregnant	68,031/ 490377 (13.8%)	19,918/ 158311 (12.5%)	17,622/ 130997 (13.4%)	16,488/ 110538 (14.9%)	6,926/ 41507 (16.6%)	7,077/ 49024 (14.4%)
Not Applicable	419,737/ 490377 (85.5%)	137,388/ 158311 (86.7%)	112,634/ 130997 (85.9%)	93,629/ 110538 (84.7%)	34,387/ 41507 (82.8%)	41,699/ 49024 (85.0%)
**Ethnicity**						
White	118,323/ 460954 (25.6%)	10,351/ 153949 (6.7%)	12,175/ 118517 (10.2%)	48,710/ 101964 (47.7%)	34,590/ 40531 (85.3%)	12,497/ 45993 (27.1%)
African background	38,171/ 460954 (8.2%)	10,612/ 153949 (6.8%)	12,824/ 118517 (10.8%)	9,894/ 101964 (9.7%)	1,097/ 40531 (2.7%)	3,744/ 45993 (8.1%)
Asian background	4,478/ 460954 (0.9%)	1,203/ 153949 (0.7%)	1,031/ 118517 (0.8%)	1,464/ 101964 (1.4%)	216/ 40531 (0.5%)	564/ 45993 (1.2%)
Mixed background	281,154/ 460954 (60.9%)	120,024/ 153949 (77.9%)	89,380/ 118517 (75.4%)	41,344/ 101964 (40.5%)	4,066/ 40531 (10.0%)	26,340/ 45993 (57.2%)
Native indigenous	18,828/ 460954 (4.0%)	11,759/ 153949 (7.6%)	3,107/ 118517 (2.62%)	552/ 101964 (0.5%)	562/ 40531 (1.3%)	2,848/ 45993 (6.1%)
**Education**						
Illiterate	22,201/ 304237 (7.3%)	8,760/ 107573 (8.1%)	8,688/ 72144 (12.0%)	2,463/ 63801 (3.8%)	659/ 29737 (2.22%)	1,631/ 30982 (5.2%)
1st to 4th incomplete grades of EF (former primary or 1st grade)	82,901/ 304237 (27.25%)	32,745/ 107573 (30.4%)	21,324/ 72144 (29.5%)	15,039/ 63801 (23.5%)	6,773/ 29737 (22.7%)	7,020/ 30982 (22.6%)
4th complete series of EF (former primary or 1st grade)	37,240/ 304237 (12.2%)	13,061/ 107573 (12.1%)	8,537/ 72144 (11.8%)	8,102/ 63801 (12.7%)	4,215/ 29737 (14.1%)	3,325/ 30982 (10.7%)
5th to 8th grade incomplete of EF (former high school or 1st grade)	72,202/ 304237 (23.7%)	27,286/ 107573 (25.3%)	15,333/ 72144 (21.25%)	14,058/ 63801 (22.0%)	7,675/ 29737 (25.8%)	7,850/ 30982 (25.3%)
Complete elementary school (former high school or 1st grade)	25,447/ 304237 (8.3%)	7,572/ 107573 (7.0%)	5,309/ 72144 (7.3%)	6,580/ 63801 (10.3%)	3,131/ 29737 (10.5%)	2,855/ 30982 (9.2%)
Incomplete high school (former high school or 2nd grade)	25,148/ 304237 (8.2%)	8,170/ 107573 (7.5%)	5,296/ 72144 (7.3%)	6,047/ 63801 (9.4%)	2,536/ 29737 (8.5%)	3,099/ 30982 (10.00%)
Complete high school (former high school or 2nd grade)	32,345/ 304237 (10.6%)	8,565/ 107573 (7.9%)	6,503/ 72144 (9.0%)	9,407/ 63801 (14.7%)	3,849/ 29737 (12.9%)	4,021/ 30982 (12.9%)
Incomplete higher education	2,304/ 304237 (0.7%)	498/ 107573 (0.4%)	395/ 72144 (0.5%)	708/ 63801 (1.1%)	328/ 29737 (1.10%)	375/ 30982 (1.2%)
Education full upper	4,449/ 304237 (1.4%)	916/ 107573 (0.8%)	759/ 72144 (1.0%)	1,397/ 63801 (2.1%)	571/ 29737 (1.9%)	806/ 30982 (2.60%)
**Elapsed Time Sting/Attendance**						
0 to 1h	151,515/ 470743 (32.1%)	30,251/ 151887 (19.9%)	34,618/ 124077 (27.9%)	48,655/ 106860 (45.5%)	20,044/ 40293 (49.7%)	17,947/ 47626 (37.6%)
1 to 3h	165,490/ 470743 (35.1%)	49,878/ 151887 (32.8%)	47,022/ 124077 (37.9%)	38,135/ 106860 (35.6%)	13,434/ 40293 (33.3%)	17,021/ 47626 (35.7%)
3 to 6h	80,480/ 470743 (17.1%)	35,075/ 151887 (23.0%)	23,525/ 124077 (18.9%)	11,265/ 106860 (10.5%)	3,554/ 40293 (8.8%)	7,061/ 47626 (14.8%)
6 to 12h	31,924/ 470743 (6.7%)	16,879/ 151887 (11.1%)	8,076/ 124077 (6.5%)	3,395/ 106860 (3.1%)	1,035/ 40293 (2.57%)	2,539/ 47626 (5.3%)
12 to 24h	21,977/ 470743 (4.67%)	10,812/ 151887 (7.1%)	5,901/ 124077 (4.7%)	2,638/ 106860 (2.4%)	973/ 40293 (2.4%)	1,653/ 47626 (3.4%)
>24h	19,357/ 470743 (4.1%)	8,992/ 151887 (5.9%)	4,935/ 124077 (3.9%)	2,772/ 106860 (2.5%)	1,253/ 40293 (3.1%)	1,405/ 47626 (2.9%)
**Location of the bite**						
Foot	233,608/ 494999 (47.1%)	85,849/ 159481 (53.8%)	63,807/ 131738 (48.4%)	44,362/ 112430 (39.4%)	17,105/ 41667 (41.0%)	22,485/ 49683 (45.2%)
Leg	101,568/ 494999 (20.5%)	37,240/ 159481 (23.3%)	20,476/ 131738 (15.5%)	23,117/ 112430 (20.5%)	8,898/ 41667 (21.3%)	11,837/ 49683 (23.8%)
Hand	59,469/ 494999 (12.0%)	12,906/ 159481 (8.0%)	16,831/ 131738 (12.7%)	17,647/ 112430 (15.7%)	6,090/ 41667 (14.6%)	5,995/ 49683 (12.0%)
Toe	34,814/ 494999 (7.0%)	9,426/ 159481 (5.9%)	11,833/ 131738 (8.9%)	7,700/ 112430 (6.8%)	2,728/ 41667 (6.5%)	3,127/ 49683 (6.2%)
Finger	34,335/ 494999 (6.9%)	5,440/ 159481 (3.4%)	10,645/ 131738 (8.0%)	11,211/ 112430 (9.9%)	3,788/ 41667 (9.0%)	3,251/ 49683 (6.5%)
Arm	10,210/ 494999 (2.06%)	2,569/ 159481 (1.6%)	2,847/ 131738 (2.1%)	2,758/ 112430 (2.4%)	1,039/ 41667 (2.4%)	997/ 49683 (2.0%)
Forearm	7,823/ 494999 (1.5%)	1,696/ 159481 (1.0%)	1,792/ 131738 (1.3%)	2,672/ 112430 (2.3%)	905/ 41667 (2.1%)	758/ 49683 (1.5%)
Head	5,695/ 494999 (1.3%)	1,766/ 159481 (1.1%)	1,661/ 131738 (1.2%)	1,294/ 112430 (1.3%)	439/ 41667 (1.0%)	535/ 49683 (1.0%)
Thigh	4,600/ 494999 (0.9%)	1,799/ 159481 (1.1%)	1,002/ 131738 (0.7%)	962/ 112430 (0.8%)	398/ 41667 (0.9%)	439/ 49683 (0.8%)
Trunk	2,877/ 494999 (0.5%)	790/ 159481 (0.5%)	844/ 131738 (0.6%)	707/ 112430 (0.6%)	277/ 41667 (0.6%)	259/ 49683 (0.5%)
**Local Manifestations**	450,985/ 492986 (91.4%)	148,511/ 157995 (94.0%)	115,675/ 131789 (87.7%)	103,426/ 112290 (92.1%)	38,432/ 41527 (92.5%)	44,941/ 49385 (91.0%)
Ache	431,965/ 451291 (95.7%)	145,094/ 148927 (97.4%)	108,584/ 115630 (93.9%)	98,865/ 103375 (95.6%)	36,206/ 38418 (94.2%)	43,216/ 44941 (96.1%)
Edema	344,160/ 449089 (76.6%)	125,679/ 148650 (84.5%)	79,297/ 114414 (69.3%)	74,341/ 102921 (72.2%)	30,071/ 38382 (78.3%)	34,772/ 44722 (77.7%)
Ecchymosis	59,955/ 442824 (13.5%)	19,641/ 146982 (13.3%)	10,607/ 112225 (9.4%)	15,324/ 101525 (15.0%)	7,908/ 38145 (20.7%)	6,475/ 43947 (14.7%)
Necrosis	7,663/ 441703 (1.7%)	2,688/ 146754 (1.8%)	1,510/ 111890 (1.3%)	1,432/ 101176 (1.4%)	1,220/ 38076 (3.2%)	813/ 43807 (1.8%)
Other locations	36,778/ 432650 (8.5%)	6,367/ 143850 (4.4%)	12,389/ 109937 (11.2%)	9,117/ 99174 (9.1%)	4,572/ 37249 (12.2%)	4,333/ 42440 (10.3%)
**Systemic manifestations**	82,097/ 473473 (17.3%)	26,325/ 152010 (17.3%)	24,432/ 125120 (19.5%)	17,185/ 108372 (15.8%)	5,490/ 40448 (13.5%)	8,665/ 47523 (18.2%)
Neuroparalytic	28,711/ 80253 (35.7%)	7,715/ 25992 (29.6%)	10,005/ 23633 (42.3%)	6,470/ 16843 (38.4%)	1,606/ 5418 (29.6%)	2,915/ 8367 (34.8%)
Hemorrhagic	19,951/ 80033 (24.9%)	9,112/ 26005 (35.0%)	5,058/ 23489 (21.5%)	3,363/ 16795 (20.0%)	994/ 5411 (18.3%)	1,424/ 8333 (17.0%)
Specify vagal (vomiting/diarrhea)	29,215/ 80092 (36.4%)	10,426/ 25973 (40.1%)	7,384/ 23473 (31.4%)	5,991/ 16787 (35.6%)	1,860/ 5419 (34.3%)	3,554/ 8440 (42.1%)
Myolytic/ hemolytic	15,341/ 79569 (19.2%)	4,807/ 25862 (18.5%)	4,507/ 23286 (19.3%)	3,138/ 16719 (18.7%)	1,182/ 5400 (21.8%)	1,707/ 8302 (20.5%)
Kidney (oliguria/anuria)	8,968/ 79396 (11.3%)	3,142/ 25841 (12.1%)	2,360/ 23206 (10.1%)	1,567/ 16672 (9.4%)	699/ 5390 (12.9%)	1,200/ 8287 (14.4%)
Other systemic	19,387/ 78321 (24.7%)	4,371/ 25313 (17.2%)	6,912/ 23074 (29.9%)	4,259/ 16497 (25.8%)	1,536/ 5321 (28.8%)	2,309/ 8116 (28.4%)
**Clotting time**						
Normal	136,584/ 223550 (61.1%)	48,290/ 72163 (66.9%)	29,548/ 54694 (54.0%)	29,497/ 50612 (58.2%)	13,884/ 21717 (63.9%)	15,365/ 24364 (63.0%)
Altered	86,966/ 223550 (38.9%)	23,873/ 72163 (33.0%)	25,146/ 54694 (45.9%)	21,115/ 50612 (41.7%)	7,833/ 21717 (36.0%)	8,999/ 24364 (36.9%)
**Snake - Type of envenomation**						
Bothropic	358,293/ 503737 (71.1%)	132,794/ 160879 (82.5%)	79,996/ 136386 (58.6%)	77,508/ 113999 (67.9%)	30,971/ 42129 (73.5%)	37,024/ 50344 (73.5%)
Crotalic	40,103/ 503737 (7.9%)	3,493/ 160879 (2.1%)	15,176/ 136386 (11.1%)	14,012/ 113999 (12.2%)	2,042/ 42129 (4.8%)	5,380/ 50344 (10.6%)
Elapidic	4,429/ 503737 (0.8%)	522/ 160879 (0.3%)	2,432/ 136386 (1.7%)	897/ 113999 (0.7%)	274/ 42129 (0.6%)	304/ 50344 (0.6%)
Laquetic	11,491/ 503737 (2.2%)	10,313/ 160879 (6.4%)	598/ 136386 (0.4%)	138/ 113999 (0.1%)	23/ 42129 (0.0%)	419/ 50344 (0.8%)
Non-Venomous Serpent	29,226/ 503737 (5.8%)	3,939/ 160879 (2.4%)	12,462/ 136386 (9.1%)	6,793/ 113999 (5.9%)	4,107/ 42129 (9.7%)	1,925/ 50344 (3.8%)
Ignored	60,195/ 503737 (11.9%)	9,818/ 160879 (6.1%)	25,722/ 136386 (18.8%)	14,651/ 113999 (12.8%)	4,712/ 42129 (11.1%)	5,292/ 50344 (10.5%)
**Case Classification**						
Mild	264,496/ 473529 (55.8%)	80,930/ 153072 (52.8%)	76,096/ 124398 (61.1%)	60,301/ 108124 (55.7%)	22,242/ 40386 (55.0%)	24,927/ 47549 (52.4%)
Moderate	174,034/ 473529 (36.7%)	62,637/ 153072 (40.9%)	40,105/ 124398 (32.2%)	38,815/ 108124 (35.9%)	14,070/ 40386 (34.8%)	18,407/ 47549 (38.7%)
Severe	34,999/ 473529 (7.3%)	9,505/ 153072 (6.3%)	8,197/ 124398 (6.5%)	9,008/ 108124 (8.3%)	4,074/ 40386 (10.0%)	4,215/ 47549 (8.8%)
**Serum therapy**	402,194/ 483406 (83.2%)	143,565/ 157504 (91.3%)	94,541/ 127097 (74.3%)	89,510/ 109102 (82.0%)	31,205/ 40831 (76.4%)	43,373/ 48872 (88.7%)
Unknown	20,331	3,375	9,289	4,897	1,298	1,472
**Local complications**	18,039/ 436315 (4.1%)	7,736/ 138758 (5.5%)	3,003/ 112566 (2.6%)	3,176/ 101775 (3.1%)	1,615/ 38470 (4.2%)	2,509/ 44746 (5.6%)
Secondary Infection	12,838/ 17788 (72.1%)	5,885/ 7672 (76.7%)	2,167/ 2946 (73.5%)	2,020/ 3101 (65.1%)	992/ 1598 (62.0%)	1,774/ 2471 (71.7%)
Extensive Necrosis	3,101/ 17373 (17.8%)	1,145/ 7532 (15.2%)	522/ 2833 (18.4%)	621/ 3020 (20.5%)	399/ 1568 (25.4%)	414/ 2420 (17.1%)
Behavioral Syndrome	2,556/ 17288 (14.7%)	1,245/ 7517 (16.5%)	251/ 2807 (8.9%)	494/ 2996 (16.4%)	157/ 1560 (10.0%)	409/ 2408 (16.9%)
Functional Deficit	2,731/ 17264 (15.8%)	1,156/ 7500 (15.4%)	420/ 2800 (15.0%)	523/ 2999 (17.4%)	282/ 1553 (18.1%)	350/ 2412 (14.5%)
Amputation	373/ 17162 (2.1%)	143/ 7463 (1.9%)	92/ 2785 (3.3%)	68/ 2961 (2.3%)	31/ 1561 (1.9%)	39/ 2392 (1.6%)
**Systemic Complications**	6,146/ 427539 (1.4%)	1,958/ 135905 (1.4%)	1,554/ 109371 (1.4%)	1,286/ 100426 (1.2%)	530/ 38095 (1.3%)	818/ 43742 (1.8%)
Renal	3,884/ 6032 (64.3%)	846/ 1928 (43.8%)	1,100/ 1515 (72.6%)	908/ 1269 (71.5%)	389/ 519 (74.9%)	641/ 801 (80.0%)
Respiratory/Acute Pulmonary Edema	1,885/ 5922 (31.8%)	650/ 1911 (34.0%)	515/ 1485 (34.6%)	396/ 1241 (31.9%)	136/ 518 (26.4%)	188/ 767 (24.5%)
Septicemia	555/ 5815 (9.5%)	248/ 1901 (13.0%)	109/ 1424 (7.6%)	102/ 1227 (8.3%)	37/ 508 (7.2%)	59/ 755 (7.8%)
Shock	1,723/ 5877 (29.3%)	901/ 1912 (47.1%)	329/ 1459 (22.5%)	261/ 1233 (21.1%)	103/ 509 (20.2%)	129/ 764 (16.8%)
**Work related envenomation**	136,536/ 427078 (31.9%)	49,228/ 135556 (36.3%)	30,638/ 108664 (28.2%)	31,952/ 100306 (31.8%)	13,119/ 38674 (33.9%)	11,599/ 43878 (26.4%)
**Evolution of the case**						
Cure	433,235/ 435474 (99.4%)	138,853/ 139612 (99.4%)	111,866/ 112613 (99.3%)	100,719/ 101083 (99.6%)	37,805/ 37926 (99.6%)	43,992/ 44240 (99.4%)
Death from envenomation	2,039/ 435474 (0.4%)	691/ 139612 (0.4%)	688/ 112613 (0.6%)	324/ 101083 (0.3%)	108/ 37926 (0.2%)	228/ 44240 (0.5%)
Death from other causes	200/ 435474 (0.0%)	68/ 139612 (0.0%)	59/ 112613 (0.0%)	40/ 101083 (0.0%)	13/ 37926 (0.0%)	20/ 44240 (0.0%)
	**Total**					
	**Complete (%)**					
Region	55.08 - Poor					
Biological sex	99.98- Excellent					
Pregnant	97.35- Excellent					
Ethnicity	91.51 - Good					
Education	60.40 - Poor					
State of residence	99.85- Excellent					
Resident Municipality	99.85- Excellent					
Occupation	52.64 - Poor					
Elapsed Time Sting/Attendance	93.45 - Good					
Location of the bite	98.27- - Excellent					
Local Manifestations	97.87- Excellent					
Ache	91.54 - Good					
Edema	91.10 - Good					
Ecchymosis	89.82 - Average					
Necrosis	89.60 - Average					
Other locations	87.76 - Average					
Other locations(specify)	6.94 - Very poor					
Systemic manifestations	93.99 - Good					
Neuroparalytic	16.95 - Very poor					
Hemorrhagic	16.90 - Very poor					
Specify vagal (vomiting/diarrhea)	16.92 - Very poor					
Myolytic/ hemolytic	16.81 - Very poor					
kidney (oliguria/anuria)	16.77 - Very poor					
Other systemic	16.54 - Very poor					
Other systemic (specify)	3.71 - Very poor					
Clotting time	44.38 - Very poor					
Case Classification	94.00 - Good					
Serum therapy	95.96- Excellent					
Local complications	86.62 - Average					
Secondary Infection	4.08 - Very poor					
Extensive Necrosis	3.98 - Very poor					
Behavioral Syndrome	3.96 - Very poor					
Functional Deficit	3.96 - Very poor					
Amputation	3.93 - Very poor					
Systemic Complications	97.99- Excellent					
Renal	1.38 - Very poor					
Respiratory/Acute Pulmonary Edema	1.36 - Very poor					
Septicemia	1.33 - Very poor					
Shock	1.35 - Very poor					
Work related envenomation	84.78 - Average					
Evolution of the case	86.45 - Average					
Date of Death	73.38 - Poor					
Closing date	95.63- Excellent					
Typing date	58.34 - Poor					

In the assessment of reliability between SINAN and the Mortality Information System (SIM), the overall Intraclass Correlation Coefficient (ICC) for fatal cases was 0.95, considered excellent. The South region had ICCs classified Good and Southeast, North, Northeast and Center-West showed lower agreement, classified as Moderate (Table 2).

**Table 2:** Inter-database reliability of fatal outcomes between SINAN and the Mortality Information System (SIM), by region. Intraclass Correlation Coefficient (ICC) values for the “death” variable comparing notifications from SINAN and records from the Mortality Information System (SIM) across Brazilian regions. Agreement categories are based on Weir’s interpretation: excellent (0.81–1.00), very good (0.61–0.80), good (0.41–0.60), fair (0.21–0.40), and poor (<0.20).

	ICC	Confiability	F	df1	df2	p	lower bound	upper bound
Total	0.95	Excellent	23	92	92	<0.01	0.9	0.97
North	0.72	Moderate	3.7	16	16	<0.01	0.26	0.9
Northeast	0.58	Moderate	3.3	16	16	0.01	-0.114	0.85
Southeast	0.7	Moderate	4.8	16	16	<0.01	0.06	0.9
South	0.87	Good	7.9	16	16	<0.01	0.65	0.95
Midwest	0.54	Moderate	2.3	16	16	0.05	-0.165	0.83

The internal consistency analysis identified logical inconsistencies in 6.8% of the total notifications. These included inconsistencies in the age format, subfields filled while parent fields were ignored, and mismatches between municipality and state codes in location-related variables (Table 3).

**Table 3:** Frequency of internal inconsistencies in snakebite notifications in Brazil (2007–2023). Types and frequencies of internal inconsistencies identified in the SINAN database, including incoherent age formatting, subfields filled despite blank parent fields, and geographic code mismatches. Percentages refer to the proportion of total records with each inconsistency.

	Brazil N = 503,737^*1*^	North N = 160,879^*1*^	Northeast N = 136,386^*1*^	Southeast N = 113,999^*1*^	South N = 42,129^*1*^	Midwest N = 50,344^*1*^
The age is not in the standard used by SINAN	10/ 503737 (0.0%)	2/ 160879 (0.0%)	4/ 136386 (0.0%)	2/ 113999 (0.0%)	2/ 42129 (0.0%)	0/ 50344 (0.0%)
A local manifestation feature but it was unfilled or ignored	1,132/ 494159 (0.2%)	500/ 158515 (0.3%)	360/ 132162 (0.2%)	155/ 112450 (0.1%)	32/ 41560 (0.0%)	85/ 49472 (0.1%)
A systemic manifestation feature but it was not fulfilled or ignored	74/ 473631 (0.0%)	23/ 152077 (0.0%)	36/ 125185 (0.0%)	5/ 108383 (0.0%)	2/ 40452 (0.0%)	8/ 47534 (0.0%)
A local complication feature but it was unfilled or ignored	9/ 436343 (0.0%)	5/ 138771 (0.0%)	1/ 112570 (0.0%)	2/ 101783 (0.0%)	1/ 38472 (0.0%)	0/ 44747 (0.0%)
A systemic complication feature but it was not fulfilled or ignored	3/ 427556 (0.0%)	0/ 135909 (0.0%)	1/ 109377 (0.0%)	2/ 100432 (0.0%)	0/ 38096 (0.0%)	0/ 43742 (0.0%)
City of service does not belong to the State of service	6,539/ 503737 (1.3%)	3,163/ 160879 (1.9%)	1,274/ 136386 (0.9%)	709/ 113999 (0.6%)	165/ 42129 (0.3%)	1,228/ 50344 (2.4%)
Municipality of residence does not belong to the State of residence	4/ 502977 (0.0%)	1/ 160281 (0.0%)	2/ 136384 (0.0%)	0/ 113990 (0.0%)	1/ 42065 (0.0%)	0/ 50257 (0.0%)

Regarding antivenom use, the study found variable adequacy depending on the snake genus. For *Bothrops*, 42.1% of cases were treated appropriately, while 10.6% received non-recommended antivenom and 11.8% were overdosed. For *Crotalus*, only 23.9% received the correct treatment, and 24.6% received inappropriate antivenom. Among *Micrurus* cases, deviations from standard dosing protocols occurred in 49.6%, and underdosing was observed in 37%. For *Lachesis*, underdosage occurred in 60.6% of cases, with correct treatment reported in only 28%. In addition, 6% of patients classified as non-venomous received antivenom unnecessarily (Table 4).

**Table 4:** Assessment of antivenom use adequacy by snake genus in Brazil (2007–2023). Classification of antivenom administration by type, dosage, and adequacy in SINAN notifications, stratified by identified snake genus. Categories include appropriate use, underdosage, overdosage, use of non-recommended antivenom, and undefined combinations.

*Bothrops* sp.	Brazil N = 358,293^*1*^	North N = 132,794^*1*^	Northeast N = 79,996^*1*^	Southeast N = 77,508^*1*^	South N = 30,971^*1*^	Midwest N = 37,024^*1*^
It was administered serum that are not used for bothropic envenomation	37,957 (10.5%)	10,086 (7.6%)	12,589 (15.7%)	8,317 (10.7%)	3,518 (11.3%)	3,447 (9.3%)
Administered serums that are used for bothropic envenomation, but in conjunction with not recommended.	3,329 (0.9%)	576 (0.4%)	1,129 (1.4%)	813 (1.0%)	125 (0.4%)	686 (1.8%)
**Antivenom**						
Adequate	150,906 (42.1%)	56,763 (42.7%)	26,942 (33.6%)	37,607 (48.5%)	14,960 (48.3%)	14,634 (39.5%)
Underdosage	26,873 (7.5%)	7,983 (6.0%)	6,850 (8.5%)	6,238 (8.0%)	3,083 (9.9%)	2,719 (7.3%)
Overdose	42,390 (11.8%)	14,145 (10.6%)	11,330 (14.1%)	7,557 (9.7%)	3,599 (11.6%)	5,759 (15.5%)
Missing Serum therapy	6,108 (1.7%)	1,473 (1.1%)	2,399 (3.0%)	1,221 (1.5%)	419 (1.3%)	596 (1.6%)
Missing Case Classification	13,903 (3.8%)	4,740 (3.5%)	4,541 (5.6%)	2,410 (3.1%)	850 (2.7%)	1,362 (3.6%)
Do not administer antivenom	11,105 (3.1%)	3,091 (2.3%)	3,597 (4.5%)	2,401 (3.1%)	960 (3.1%)	1,056 (2.8%)
Administered antivenin just doesn’t know which or how much	107,008 (29.8%)	44,599 (33.5%)	24,337 (30.4%)	20,074 (25.9%)	7,100 (22.9%)	10,898 (29.4%)
***Crotalus* sp.**	**Brazil** **N = 40,103** ^***1***^	**North** **N = 3,493** ^***1***^	**Northeast** **N = 15,176** ^***1***^	**Southeast** **N = 14,012** ^***1***^	**South** **N = 2,042** ^***1***^	**Midwest** **N = 5,380** ^***1***^
It was Administered serum that are not used for *Crotalus* envenomation	9,866 (24.6%)	1,037 (29.6%)	4,189 (27.6%)	3,209 (22.9%)	647 (31.6%)	784 (14.5%)
Administered serums that are used for *Crotalus* envenomation, however in conjunction with not recommended.	25,409 (63.3%)	1,755 (50.2%)	9,333 (61.5%)	9,360 (66.8%)	1,207 (59.1%)	3,754 (69.7%)
Antivenom						
Adequate	9,581 (23.8%)	550 (15.7%)	2,752 (18.1%)	4,262 (30.4%)	574 (28.1%)	1,443 (26.8%)
Underdosage	9,897 (24.6%)	1,236 (35.3%)	3,816 (25.1%)	2,750 (19.6%)	588 (28.8%)	1,507 (28.%)
Overdose	3,298 (8.2%)	221 (6.3%)	1,312 (8.6%)	1,154 (8.2%)	108 (5.2%)	503 (9.3%)
Missing Serum therapy	1,129 (2.8%)	72 (2.0%)	585 (3.8%)	297 (2.1%)	73 (3.5%)	102 (1.9%)
Missing Case Classification	1,504 (3.7%)	137 (3.9%)	759 (5.0%)	372 (2.6%)	34 (1.6%)	202 (3.7%)
Do not administer antivenom	2,931 (7.3%)	177 (5.07%)	1,199 (7.9%)	1,178 (8.4%)	205 (10.0%)	172 (3.2%)
Administered antivenin just doesn’t know which or how much	11,763 (29.3%)	1,100 (31.4%)	4,753 (31.3%)	3,999 (28.5%)	460 (22.5%)	1,451 (26.9%)
***Micrurus* sp.**	**Brazil** **N = 4,429** ^***1***^	**North** **N = 522** ^***1***^	**Northeast** **N = 2,432** ^***1***^	**Southeast** **N = 897** ^***1***^	**South** **N = 274** ^***1***^	**Midwest** **N = 304** ^***1***^
It was Administered serum that are not used for Elapidic envenomation	2,198 (49.6%)	264 (50.5%)	1,176/ 2432 (48.3%)	521/ 897 (58.0%)	108/ 274 (39.4%)	129/ 304 (42.4%)
Administered serums that are used for Elapidic envenomation, however in conjunction with not recommended.	456 (10.3%)	156 (29.8%)	172/ 2432 (7.0%)	74/ 897 (8.2%)	19/ 274 (6.9%)	35/ 304 (11.5%)
Antivenom						
Adequate	1,321 (29.8%)	151 (28.9%)	764 (31.4%)	239 (26.6%)	96 (35.0%)	71 (23.3%)
Underdosage	1,639 (37.0%)	236 (45.3%)	812 (33.3%)	334 (37.2%)	110 (40.3%)	147 (48.3%)
Overdose	77 (1.7%)	1 (0.1%)	66 (2.7%)	4 (0.4%)	1 (0.3%)	5 (1.6%)
Missing Serum therapy	225 (5.0%)	12 (2.3%)	159 (6.5%)	37 (4.1%)	3 (1.0%)	14 (4.6%)
Missing Case Classification	205 (4.6%)	19 (3.6%)	127 (5.2%)	37 (4.1%)	8 (2.9%)	14 (4.6%)
Do not administer antivenom	737 (16.6%)	52 (9.9%)	392 (16.1%)	209 (23.3%)	46 (16.7%)	38 (12.5%)
Administered antivenin just doesn’t know which or how much	225 (5.0%)	51 (9.7%)	112 (4.6%)	37 (4.1%)	10 (3.6%)	15 (4.9%)
***Lachesis* sp.**	**Brazil** **N = 11,491** ^***1***^	**North** **N = 10,313** ^***1***^	**Northeast** **N = 598** ^***1***^	**Southeast** **N = 138** ^***1***^	**South** **N = 23** ^***1***^	**Midwest** **N = 419** ^***1***^
It was Administered serum that are not used for Laquetic envenomation	2,521 (21.9%)	1,997 (19.3%)	306 (51.1%)	112 (81.1%)	23 (100.0%)	83 (19.8%)
Administered serums that are used for Laquetic envenomation, but in conjunction with not recommended.	1,847 (16.0%)	1,575 (15.2%)	133 (22.2%)	57 (41.3%)	8 (34.7%)	74 (17.6%)
Antivenom						
Adequate	3,219 (28.0%)	3,016 (29.2%)	108 (18.0%)	6 (4.3%)	0 (0.0%)	89 (21.2%)
Underdosage	6,959 (60.5%)	6,244 (60.5%)	326 (54.5%)	86 (62.2%)	10 (43.4%)	293 (69.9%)
Overdose	0 (0.0%)	0 (0.0%)	0 (0.0%)	0 (0.0%)	0 (0.0%)	0 (0.0%)
Missing Serum therapy	150 (1.3%)	105 (1.0%)	29 (4.8%)	9 (6.5%)	3 (13.0%)	4 (0.9%)
Missing Case Classification	511 (4.4%)	466 (4.5%)	29 (4.8%)	4 (2.9%)	0 (0.0%)	12 (2.8%)
Do not administer antivenom	245 (2.1%)	158 (1.5%)	57 (9.5%)	16 (11.5%)	4 (17.3%)	10 (2.3%)
Administered antivenin just doesn’t know which or how much	407 (3.5%)	324 (3.1%)	49 (8.1%)	17 (12.3%)	6 (26.0%)	11 (2.6%)
**Non-venomous**	**Brazil** **N = 29,226** ^***1***^	**North** **N = 3,939** ^***1***^	**Northeast** **N = 12,462** ^***1***^	**Southeast** **N = 6,793** ^***1***^	**South** **N = 4,107** ^***1***^	**Midwest** **N = 1,925** ^***1***^
Envenomation classified as non-venomous but treated with saline.	1,755 (6.0%)	404 (10.2%)	687 (5.5%)	300 (4.4%)	164 (3.9%)	200 (10.3%)

## Discussion

This study assessed the quality of snakebite envenoming (SBE) notifications in Brazil over a 17-year period, focusing on completeness, internal consistency, and reliability across the national surveillance system. In line with prior evaluations of the Brazilian Notifiable Diseases Information System (SINAN), variables such as age, sex, and municipality of residence showed excellent completeness [17–20]. However, key equity-related variables — including education, occupation, and race/ethnicity — remain poorly filled, especially in regions with greater social vulnerability. This restricts the capacity of the system to capture health disparities or monitor the differentiated impact of snakebites on vulnerable populations. Previous analyses of SINAN data for other conditions, such as leprosy and dengue, have identified similar patterns [19,21], suggesting a systemic challenge in integrating social determinants into surveillance practices.

While some core sociodemographic and outcome variables demonstrated high completeness and agreement, the overall quality of snakebite surveillance data remains limited by persistent gaps — particularly in clinical, therapeutic, and socioeconomic fields. These weaknesses hinder the ability of the system to produce actionable information for antivenom allocation and health planning.

Clinical fields, particularly those detailing systemic manifestations, complications, and clotting time, showed high proportions of missing or “ignored” values. These results echo findings from other national studies on envenomation [22,23], and highlight a disconnection between clinical care and surveillance reporting. This is particularly relevant for variables that are central to assessing severity and treatment adequacy — including the type and dosage of antivenom used. The low completeness in these fields may reflect a combination of operational barriers, unfamiliarity with reporting protocols, and a lack of integration between medical and epidemiological responsibilities within health teams. Although there are other notification systems [24] applied to health in other countries, none have proven to be as complete and comprehensive as SINAN, even with the observed filling limitations.

The internal consistency analysis revealed relatively few but meaningful inconsistencies, including incoherent age formatting and contradictions between hierarchical fields (e.g., presence of subfields without corresponding parent fields). Although these inconsistencies affected a small proportion of notifications, they are important quality indicators — especially because they suggest difficulties in the design or understanding of the notification forms. Similar issues have been documented in evaluations of completeness and consistency for tuberculosis, syphilis, and maternal health data in Brazil [14,25,26].

The high reliability observed between SINAN and the Mortality Information System (SIM) for fatal outcomes (ICC = 0.95) is encouraging, but regional discrepancies remain. In regions with lower agreement, such as the Northeast and Center-West, interoperability between information systems may be limited, or deaths may be underreported in one or both systems. These challenges have been previously identified in national audits of cause-of-death reporting and may be particularly relevant for conditions like SBE, which often occur in remote or under-resourced areas [14,27,28].

A particularly critical point is the observed inadequacy in antivenom use — especially in cases involving *Crotalus*, *Lachesis*, and *Micrurus*. The finding that a significant amount of Micrurus and Lachesis cases were inappropriately treated is alarming, and it points to major gaps in clinical practice and antivenom availability. Previous studies have shown that underdosage, use of incorrect antivenoms, and empirical treatment are more frequent in these scenarios, largely due to clinical uncertainty and access issues [29,30]. These patterns raise concerns about the consequences of fragmented training and resource distribution in regions with high SBE incidence.

The North region — where almost half of the country’s snakebite cases occur — concentrates many of the weaknesses identified. Studies have consistently reported barriers to timely notification and appropriate case management in this area, including high turnover of professionals, limited internet and transportation infrastructure, and underfinancing of remote base units [10,31,32]. These challenges are particularly acute in territories served by the Indigenous Health Subsystem. Indigenous communities face higher risk of snakebites and mortality [12,33,34], but often remain underrepresented in official data due to incomplete or absent reporting. Analyses of health service provision in Indigenous districts have shown that the absence of antivenom in base units — combined with cultural mismatches in care — results in preventable delays and adverse outcomes [12,35–37].

Decentralization of antivenom and improved notification capacity have been identified in recent literature as feasible and effective strategies to reduce inequities in SBE care [10,38]. However, this requires not only logistical investments (e.g., cold chain infrastructure and safe transport), but also the revision of training models and notification workflows to ensure adequate use of the SINAN platform in remote and intercultural contexts. Recent reviews of antivenom access in Latin America suggest that decentralization efforts, when paired with robust training and adapted protocols, can lead to substantial reductions in time-to-treatment and complications [10,39,40].

This study used secondary data exclusively from SINAN and SIM and could not validate the accuracy of entries through direct clinical record review. Although internal consistency checks and inter-database reliability provide insights into data quality, they do not reveal the underlying causes of poor completeness or discordance. Considering the next steps to improve the quality of data reporting in the Brazilian snakebite surveillance system, this research generates a set of scripts, which can be used by the national coordination to generate quality reports. Furthermore, the analysis of antivenom use was limited by the incompleteness of treatment-related fields, which restricts the generalizability of some interpretations.

## Conclusions

The findings of this study confirm that key dimensions of snakebite notification quality in Brazil remain limited, particularly in the North and Northeast regions and among vulnerable populations such as Indigenous communities. While overall fatal outcome reporting between systems is reliable, persistent gaps in the recording of clinical details, treatment regimens, and social variables compromise the utility of the data for health planning and equity monitoring. Strengthening the completeness and consistency of notification forms — especially in remote areas — is essential for improving national surveillance. Enhancing training, decentralizing antivenom to qualified units, and promoting intercultural competence in reporting are concrete steps that align with Brazil’s commitment to reducing snakebite burden as a neglected tropical disease.

## Supporting information

S1 Table Epidemiological profile of reported snakebite envenoming cases in Brazil by region (2007–2023). Distribution of snakebite envenoming cases notified in the SINAN system between 2007 and 2023, stratified by Brazilian macroregions. Variables include year of occurrence, age, sex, pregnancy status, ethnicity, education level, time to attendance, anatomical site of bite, local and systemic manifestations, clotting time, snake genus, case classification, serum therapy, complications, occupational exposure, and outcomes. Percentages represent the proportion of valid responses for each category within regions.
(DOCX)

S2 Table Completeness of selected variables in snakebite envenoming notifications in Brazil (2007–2023), by region. Completeness percentages for key sociodemographic, clinical, and treatment-related variables reported in SINAN, stratified by Brazilian geographic region. Classification of completeness levels follows Romero and Cunha’s criteria: excellent (≥95%), good (90–94.9%), regular (80–89.9%), poor (50–79.9%), and very poor (<50%).
(DOCX)

S1 Fig Geographic distribution of reported snakebite envenoming cases in Brazil (2007–2023), by municipality and trends in death reporting according to information systems (SINAN) across years. Spatial distribution of snakebite envenoming notifications across Brazilian municipalities based on SINAN data from 2007 to 2023. Higher case densities are observed in the North region, particularly in Amazonian states. Municipalities with no registered cases are shown in white. A – Bothrops; B – Crotalus; C – Micrurus; D – Lachesis; E – Non-venomous snakes. In addition, a heatmap illustrates the temporal trends of snakebite notifications across Brazilian states during the study period. Source of shapefiles: Brazilian Institute of Geography and Statistics (IBGE), 2023. Shapefiles are available under the Creative Commons Attribution 4.0 License (CC BY 4.0): https://www.ibge.gov.br/geociencias/organizacao-do-territorio/malhas-territoriais/15774-malhas.html?=&t=acesso-ao-produto.
(DOCX)
