# Supplementary material for: Assessing territorial disparities in snakebite surveillance data in Brazil: Implications for public health
Source: PLoS Negl Trop Dis. 2026 Jan 16;20(1):e0013873. doi: 10.1371/journal.pntd.0013873 (PMC12810854; doi:10.1371/journal.pntd.0013873)
Supplement: S1 Table — Distribution of snakebite envenoming cases notified in the SINAN system between 2007 and 2023, stratified by Brazilian macroregions. Variables include year of occurrence, age, sex, pregnancy status, ethnicity, education level, time to attendance, anatomical site of bite, local and systemic manifestations, clotting time, snake genus, case classification, serum therapy, complications, occupational exposure, and outcomes. Percentages represent the proportion of valid responses for each category within regions. (DOCX) [file pntd.0013873.s001.docx]

**Supplementary Table 1**

| **Characteristic** | **Overall** | **Norte** | **Nordeste** | **Sudeste** | **Sul** | **Centro-Oeste** |
| --- | --- | --- | --- | --- | --- | --- |
|  | N = 503,737*^1^* | N = 160,879*^1^* | N = 136,386*^1^* | N = 113,999*^1^* | N = 42,129*^1^* | N = 50,344*^1^* |
| **Year** |  |  |  |  |  |  |
| 2007 | 26,560 (5.2%) | 7,902 (4.9%) | 6,796 (4.9%) | 6,564 (5.7%) | 3,029 (7.1%) | 2,269 (4.5%) |
| 2008 | 27,787 (5.5%) | 8,341 (5.1%) | 6,934 (5.0%) | 6,952 (6.1%) | 2,764 (6.5%) | 2,796 (5.5%) |
| 2009 | 29,765 (5.9%) | 9,148 (5.6%) | 8,359 (6.1%) | 6,315 (5.5%) | 2,999 (7.1%) | 2,944 (5.8%) |
| 2010 | 29,657 (5.8%) | 9,227 (5.7%) | 8,183 (6.0%) | 6,388 (5.6%) | 2,717 (6.4%) | 3,142 (6.2%) |
| 2011 | 30,142 (5.9%) | 9,096 (5.6%) | 7,945 (5.8%) | 7,301 (6.4%) | 2,596 (6.1%) | 3,204 (6.3%) |
| 2012 | 28,336 (5.6%) | 8,919 (5.5%) | 6,808 (4.9%) | 7,102 (6.2%) | 2,422 (5.7%) | 3,085 (6.1%) |
| 2013 | 27,390 (5.4%) | 9,521 (5.9%) | 6,118 (4.4%) | 6,785 (5.9%) | 2,251 (5.3%) | 2,715 (5.3%) |
| 2014 | 26,209 (5.2%) | 9,469 (5.8%) | 5,889 (4.3%) | 5,746 (5.0%) | 2,352 (5.5%) | 2,753 (5.4%) |
| 2015 | 27,167 (5.3%) | 9,062 (5.6%) | 7,020 (5.2%) | 5,766 (5.0%) | 2,409 (5.7%) | 2,910 (5.7%) |
| 2016 | 26,589 (5.2%) | 8,783 (5.4%) | 7,103 (5.2%) | 5,372 (4.7%) | 2,392 (5.6%) | 2,939 (5.8%) |
| 2017 | 28,738 (5.7%) | 8,898 (5.5%) | 7,261 (5.3%) | 6,903 (6.0%) | 2,542 (6.0%) | 3,134 (6.2%) |
| 2018 | 30,476 (6.0%) | 9,798 (6.0%) | 7,904 (5.8%) | 7,556 (6.6%) | 2,396 (5.6%) | 2,822 (5.6%) |
| 2019 | 34,192 (6.7%) | 11,102 (6.9%) | 9,867 / (7.2%) | 7,476 (6.5%) | 2,452 (5.8%) | 3,295 (6.5%) |
| 2020 | 33,838 (6.7%) | 10,709 (6.6%) | 10,194 (7.4%) | 7,488 (6.5%) | 2,267 (5.3%) | 3,180 (6.3%) |
| 2021 | 33,157 (6.5%) | 10,689 (6.6%) | 10,265 (7.5%) | 6,882 (6.0%) | 2,137 (5.0%) | 3,184 (6.3%) |
| 2022 | 31,136 (6.1%) | 10,423 (6.4%) | 9,478 (6.9%) | 6,459 (5.6%) | 2,017 (4.7%) | 2,759 (5.4%) |
| 2023 | 32,598 (6.4%) | 9,792 (6.0%) | 10,262 (7.5%) | 6,944 (6.0%) | 2,387 (5.6%) | 3,213 (6.3%) |
| **Age** | 35, 19 | 32, 18 | 35, 19 | 39, 19 | 39, 19 | 37, 19 |
| **Biological sex** |  |  |  |  |  |  |
| Male | 386,466 / 503,648 (76.7%) | 126,611 / 160,864 (78.7%) | 102,535/136,353 (75.2%) | 87,030 / 113,973 (76.3%) | 31,851/ 42,126 (75.6%) | 38,439 / 50,332 (76.3%) |
| Female | 117,182 / 503,648 (23.2%) | 34,253 / 160,864 (21.2%) | 33,818 / 136,353 (24.8%) | 26,943 / 113,973 (23.6%) | 10,275/ 42,126 (24.3%) | 11,893 / 50,332 (23.6%) |
| Unknown | 89 | 15 | 33 | 26 | 3 | 12 |
| **Pregnancy data** |  |  |  |  |  |  |
| 1st Quarter | 605 / 490377 (0.1%) | 222 / 158311 (0.1%) | 144 / 130997 (0.1%) | 104 / 110538 (0.0%) | 77 / 41507 (0.1%) | 58 / 49024 (0.1%) |
| 2nd Quarter | 944 / 490377 (0.1%) | 347 / 158311 (0.2%) | 262 / 130997 (0.2%) | 164 / 110538 (0.1.%) | 75 / 41507 (0.1%) | 96 / 49024 (0.2%) |
| 3nd Quarter | 543 / 490377 (0.1%) | 222 / 158311 (0.1%) | 149 / 130997 (0.1%) | 88 / 110538 (0.0%) | 28 / 41507 (0.0%) | 56 / 49024 (0.1%) |
| Gestational Age Unknown | 517 / 490377 (0.1%) | 214 / 158311 (0.1%) | 186 / 130997 (0.1%) | 65 / 110538 (0.0%) | 14 / 41507 (0.0%) | 38 / 49024 (0.0%) |
| Not pregnant | 68,031 / 490377 (13.8%) | 19,918 / 158311 (12.5%) | 17,622 / 130997 (13.4%) | 16,488 / 110538 (14.9%) | 6,926 / 41507 (16.6%) | 7,077 / 49024 (14.4%) |
| Not Applicable | 419,737 / 490377 (85.5%) | 137,388 / 158311 (86.7%) | 112,634/ 130997 (85.9%) | 93,629 / 110538 (84.7%) | 34,387 / 41507 (82.8%) | 41,699 / 49024 (85.0%) |
| **Ethnicity** |  |  |  |  |  |  |
| White | 118,323 / 460954 (25.6%) | 10,351 / 153949 (6.7%) | 12,175 / 118517 (10.2%) | 48,710 / 101964 (47.7%) | 34,590 / 40531 (85.3%) | 12,497 / 45993 (27.1%) |
| African background | 38,171 / 460954 (8.2%) | 10,612 / 153949 (6.8%) | 12,824 / 118517 (10.8%) | 9,894 / 101964 (9.7%) | 1,097 / 40531 (2.7%) | 3,744 / 45993 (8.1%) |
| Asian background | 4,478 / 460954 (0.9%) | 1,203 / 153949 (0.7%) | 1,031 / 118517 (0.8%) | 1,464 / 101964 (1.4%) | 216 / 40531 (0.5%) | 564 / 45993 (1.2%) |
| Mixed background | 281,154 / 460954 (60.9%) | 120,024 / 153949 (77.9%) | 89,380 / 118517 (75.4%) | 41,344 / 101964 (40.5%) | 4,066 / 40531 (10.0%) | 26,340 / 45993 (57.2%) |
| Native indigenous | 18,828 / 460954 (4.0%) | 11,759 / 153949 (7.6%) | 3,107 / 118517 (2.6%) | 552 / 101964 (0.5%) | 562 / 40531 (1.3%) | 2,848 / 45993 (6.1%) |
| **Education** |  |  |  |  |  |  |
| Illiterate | 22,201 / 304237 (7.3%) | 8,760 / 107573 (8.1%) | 8,688 / 72144 (12.0%) | 2,463 / 63801 (3.8%) | 659 / 29737 (2.2%) | 1,631 / 30982 (5.2%) |
| 1st to 4th incomplete grades of EF (former primary or 1st grade) | 82,901 / 304237 (27.3%) | 32,745 / 107573 (30.4%) | 21,324 / 72144 (29.5%) | 15,039 / 63801 (23.5%) | 6,773 / 29737 (22.7%) | 7,020 / 30982 (22.6%) |
| 4th complete series of EF (former primary or 1st grade) | 37,240 / 304237 (12.2%) | 13,061 / 107573 (12.1%) | 8,537 / 72144 (11.8%) | 8,102 / 63801 (12.7%) | 4,215 / 29737 (14.1%) | 3,325 / 30982 (10.7%) |
| 5th to 8th grade incomplete of EF (former high school or 1st grade) | 72,202 / 304237 (23.7%) | 27,286 / 107573 (25.3%) | 15,333 / 72144 (21.5%) | 14,058 / 63801 (22.0%) | 7,675 / 29737 (25.8%) | 7,850 / 30982 (25.3%) |
| Complete elementary school (former high school or 1st grade) | 25,447 / 304237 (8.3%) | 7,572 / 107573 (7.0%) | 5,309 / 72144 (7.3%) | 6,580 / 63801 (10.3%) | 3,131 / 29737 (10.5%) | 2,855 / 30982 (9.2%) |
| Incomplete high school (former high school or 2nd grade) | 25,148 / 304237 (8.2%) | 8,170 / 107573 (7.5%) | 5,296 / 72144 (7.3%) | 6,047 / 63801 (9.4%) | 2,536 / 29737 (8.5%) | 3,099 / 30982 (10.0%) |
| Complete high school (former high school or 2nd grade) | 32,345 / 304237 (10.6%) | 8,565 / 107573 (7.9%) | 6,503 / 72144 (9.0%) | 9,407 / 63801 (14.7%) | 3,849 / 29737 (12.9%) | 4,021 / 30982 (12.9%) |
| Incomplete higher education | 2,304 / 304237 (0.7%) | 498 / 107573 (0.4%) | 395 / 72144 (0.5%) | 708 / 63801 (1.1%) | 328 / 29737 (1.1%) | 375 / 30982 (1.2%) |
| Education full upper | 4,449 / 304237 (1.4%) | 916 / 107573 (0.8%) | 759 / 72144 (1.0%) | 1,397 / 63801 (2.1%) | 571 / 29737 (1.9%) | 806 / 30982 (2.6%) |
| **Elapsed Time Sting/Attendance** |  |  |  |  |  |  |
| 0 to 1h | 151,515 / 470743 (32.1%) | 30,251 / 151887 (19.9%) | 34,618 / 124077 (27.9%) | 48,655 / 106860 (45.5%) | 20,044 / 40293 (49.7%) | 17,947 / 47626 (37.6%) |
| 1 to 3h | 165,490 / 470743 (35.1%) | 49,878 / 151887 (32.8%) | 47,022 / 124077 (37.9%) | 38,135 / 106860 (35.6%) | 13,434 / 40293 (33.3%) | 17,021 / 47626 (35.7%) |
| 3 to 6h | 80,480 / 470743 (17.1%) | 35,075 / 151887 (23.0%) | 23,525 / 124077 (18.9%) | 11,265 / 106860 (10.5%) | 3,554 / 40293 (8.8%) | 7,061 / 47626 (14.8%) |
| 6 to 12h | 31,924 / 470743 (6.7%) | 16,879 / 151887 (11.1%) | 8,076 / 124077 (6.5%) | 3,395 / 106860 (3.1%) | 1,035 / 40293 (2.5%) | 2,539 / 47626 (5.3%) |
| 12 to 24h | 21,977 / 470743 (4.6%) | 10,812 / 151887 (7.1%) | 5,901 / 124077 (4.7%) | 2,638 / 106860 (2.4%) | 973 / 40293 (2.4%) | 1,653 / 47626 (3.4%) |
| >24h | 19,357 / 470743 (4.1%) | 8,992 / 151887 (5.9%) | 4,935 / 124077 (3.9%) | 2,772 / 106860 (2.5%) | 1,253 / 40293 (3.1%) | 1,405 / 47626 (2.9%) |
| **Location of the bite** |  |  |  |  |  |  |
| Foot | 233,608 / 494999 (47.1%) | 85,849 / 159481 (53.8%) | 63,807 / 131738 (48.4%) | 44,362 / 112430 (39.4%) | 17,105 / 41667 (41.0%) | 22,485 / 49683 (45.2%) |
| Leg | 101,568 / 494999 (20.5%) | 37,240 / 159481 (23.3%) | 20,476 / 131738 (15.5%) | 23,117 / 112430 (20.5%) | 8,898 / 41667 (21.3%) | 11,837 / 49683 (23.8%) |
| Hand | 59,469 / 494999 (12.0%) | 12,906 / 159481 (8.0%) | 16,831 / 131738 (12.7%) | 17,647 / 112430 (15.7%) | 6,090 / 41667 (14.6%) | 5,995 / 49683 (12.0%) |
| Toe | 34,814 / 494999 (7.0%) | 9,426 / 159481 (5.9%) | 11,833 / 131738 (8.9%) | 7,700 / 112430 (6.8%) | 2,728 / 41667 (6.5%) | 3,127 / 49683 (6.2%) |
| Finger | 34,335 / 494999 (6.9%) | 5,440 / 159481 (3.4%) | 10,645 / 131738 (8.0%) | 11,211 / 112430 (9.9%) | 3,788 / 41667 (9.0%) | 3,251 / 49683 (6.5%) |
| Arm | 10,210 / 494999 (2.0%) | 2,569 / 159481 (1.6%) | 2,847 / 131738 (2.1%) | 2,758 / 112430 (2.4%) | 1,039 / 41667 (2.4%) | 997 / 49683 (2.0%) |
| Forearm | 7,823 / 494999  (1.5%) | 1,696 / 159481 (1.0%) | 1,792 / 131738 (1.3%) | 2,672 / 112430 (2.3%) | 905 / 41667 (2.1%) | 758 / 49683 (1.5%) |
| Head | 5,695 / 494999  (1.1%) | 1,766 / 159481 (1.1%) | 1,661 / 131738 (1.2%) | 1,294 / 112430 (1.1%) | 439 / 41667 (1.0%) | 535 / 49683 (1.0%) |
| Thigh | 4,600 / 494999  (0.9%) | 1,799 / 159481 (1.1%) | 1,002 / 131738 (0.7%) | 962 / 112430 (0.8%) | 398 / 41667 (0.9%) | 439 / 49683 (0.8%) |
| Trunk | 2,877 / 494999  (0.5%) | 790 / 159481 (0.5%) | 844 / 131738 (0.6%) | 707 / 112430 (0.6%) | 277 / 41667 (0.6%) | 259 / 49683 (0.5%) |
| **Local Manifestations** | 450,985 / 492986 (91.4%) | 148,511 / 157995 (94.0%) | 115,675 / 131789 (87.7%) | 103,426 / 112290 (92.1%) | 38,432 / 41527 (92.5%) | 44,941 / 49385 (91.0%) |
| Ache | 431,965 / 451291 (95.7%) | 145,094 / 148927 (97.4%) | 108,584 / 115630 (93.9%) | 98,865 / 103375 (95.6%) | 36,206 / 38418 (94.2%) | 43,216 / 44941 (96.1%) |
| Edema | 344,160 / 449089 (76.6%) | 125,679 / 148650 (84.5%) | 79,297 / 114414 (69.3%) | 74,341 / 102921 (72.2%) | 30,071 / 38382 (78.3%) | 34,772 / 44722 (77.7%) |
| Ecchymosis | 59,955 / 442824 (13.5%) | 19,641 / 146982 (13.3%) | 10,607 / 112225 (9.4%) | 15,324 / 101525 (15.0%) | 7,908 / 38145 (20.7%) | 6,475 / 43947 (14.7%) |
| Necrosis | 7,663 / 441703 (1.7%) | 2,688 / 146754 (1.8%) | 1,510 / 111890 (1.3%) | 1,432 / 101176 (1.4%) | 1,220 / 38076 (3.2%) | 813 / 43807 (1.8%) |
| Other locations | 36,778 / 432650 (8.5%) | 6,367 / 143850 (4.4%) | 12,389 / 109937 (11.2%) | 9,117 / 99174 (9.1%) | 4,572 / 37249 (12.2%) | 4,333 / 42440 (10.2%) |
| **Systemic manifestations** | 82,097 / 473473 (17.3%) | 26,325 / 152010 (17.3%) | 24,432 / 125120 (19.5%) | 17,185 / 108372 (15.8%) | 5,490 / 40448 (13.5%) | 8,665 / 47523 (18.2%) |
| Neuroparalytic | 28,711 / 80253 (35.7%) | 7,715 / 25992 (29.6%) | 10,005 / 23633 (42.3%) | 6,470 / 16843 (38.4%) | 1,606 / 5418 (29.6%) | 2,915 / 8367 (34.8%) |
| Hemorrhagic | 19,951 / 80033 (24.9%) | 9,112 / 26005 (35.0%) | 5,058 / 23489 (21.5%) | 3,363 / 16795 (20.0%) | 994 / 5411 (18.3%) | 1,424 / 8333 (17.0%) |
| Specify vagal (vomiting/diarrhea) | 29,215 / 80092 (36.4%) | 10,426 / 25973 (40.1%) | 7,384 / 23473 (31.4%) | 5,991 / 16787 (35.6%) | 1,860 / 5419 (34.3%) | 3,554 / 8440 (42.1%) |
| Myolytic / hemolytic | 15,341 / 79569 (19.2%) | 4,807 / 25862 (18.5%) | 4,507 / 23286 (19.3%) | 3,138 / 16719 (18.7%) | 1,182 / 5400 (21.8%) | 1,707 / 8302 (20.5%) |
| Kidney (oliguria/anuria) | 8,968 / 79396 (11.3%) | 3,142 / 25841 (12.1%) | 2,360 / 23206 (10.1%) | 1,567 / 16672 (9.4%) | 699 / 5390 (12.9%) | 1,200 / 8287 (14.4%) |
| Other systemic | 19,387 / 78321 (24.7%) | 4,371 / 25313 (17.2%) | 6,912 / 23074 (29.9%) | 4,259 / 16497 (25.8%) | 1,536 / 5321 (28.8%) | 2,309 / 8116 (28.4%) |
| **Clotting time** |  |  |  |  |  |  |
| Normal | 136,584 / 223550 (61.1%) | 48,290 / 72163 (66.9%) | 29,548 / 54694 (54.0%) | 29,497 / 50612 (58.2%) | 13,884 / 21717 (63.9%) | 15,365 / 24364 (63.0%) |
| Altered | 86,966 / 223550 (38.9%) | 23,873 / 72163 (33.0%) | 25,146 / 54694 (45.9%) | 21,115 / 50612 (41.7%) | 7,833 / 21717 (36.0%) | 8,999 / 24364 (36.9%) |
| **Snake - Type of envenomation** |  |  |  |  |  |  |
| Bothropic | 358,293 / 503737 (71.1%) | 132,794 / 160879 (82.5%) | 79,996 / 136386 (58.6%) | 77,508 / 113999 (67.9%) | 30,971 / 42129 (73.5%) | 37,024 / 50344 (73.5%) |
| Crotalic | 40,103 / 503737 (7.9%) | 3,493 / 160879 (2.1%) | 15,176 / 136386 (11.1%) | 14,012 / 113999 (12.2%) | 2,042 / 42129 (4.8%) | 5,380 / 50344 (10.6%) |
| Elapidic | 4,429 / 503737  (0.8%) | 522 / 160879 (0.3%) | 2,432 / 136386 (1.7%) | 897 / 113999 (0.7%) | 274 / 42129 (0.6%) | 304 / 50344 (0.6%) |
| Laquetic | 11,491 / 503737 (2.2%) | 10,313 / 160879 (6.4%) | 598 / 136386 (0.4%) | 138 / 113999 (0.1%) | 23 / 42129 (0.0%) | 419 / 50344 (0.8%) |
| Non-Venomous Serpent | 29,226 / 503737 (5.8%) | 3,939 / 160879 (2.4%) | 12,462 / 136386 (9.1%) | 6,793 / 113999 (5.9%) | 4,107 / 42129 (9.7%) | 1,925 / 50344 (3.8%) |
| Ignored | 60,195 / 503737 (11.9%) | 9,818 / 160879 (6.1%) | 25,722 / 136386 (18.8%) | 14,651 / 113999 (12.8%) | 4,712 / 42129 (11.1%) | 5,292 / 50344 (10.5%) |
| **Case Classification** |  |  |  |  |  |  |
| Mild | 264,496 / 473529 (55.8%) | 80,930 / 153072 (52.8%) | 76,096 / 124398 (61.1%) | 60,301 / 108124 (55.7%) | 22,242 / 40386 (55.0%) | 24,927 / 47549 (52.4%) |
| Moderate | 174,034 / 473529 (36.7%) | 62,637 / 153072 (40.9%) | 40,105 / 124398 (32.2%) | 38,815 / 108124 (35.9%) | 14,070 / 40386 (34.8%) | 18,407 / 47549 (38.7%) |
| Severe | 34,999 / 473529 (7.3%) | 9,505 / 153072 (6.2%) | 8,197 / 124398 (6.5%) | 9,008 / 108124 (8.3%) | 4,074 / 40386 (10.0%) | 4,215 / 47549 (8.8%) |
| **Serum therapy** | 402,194 / 483406 (83.2%) | 143,565 / 157504 (91.2%) | 94,541 / 127097 (74.3%) | 89,510 / 109102 (82.0%) | 31,205 / 40831 (76.4%) | 43,373 / 48872 (88.7%) |
| Unknown | 20,331 | 3,375 | 9,289 | 4,897 | 1,298 | 1,472 |
| **Local complications** | 18,039 / 436315 (4.1%) | 7,736 / 138758 (5.5%) | 3,003 / 112566 (2.6%) | 3,176 / 101775 (3.1%) | 1,615 / 38470 (4.2%) | 2,509 / 44746 (5.6%) |
| Secondary Infection | 12,838 / 17788 (72.1%) | 5,885 / 7672 (76.7%) | 2,167 / 2946 (73.5%) | 2,020 / 3101 (65.1%) | 992 / 1598 (62.0%) | 1,774 / 2471 (71.7%) |
| Extensive Necrosis | 3,101 / 17373 (17.8%) | 1,145 / 7532 (15.2%) | 522 / 2833 (18.4%) | 621 / 3020 (20.5%) | 399 / 1568 (25.4%) | 414 / 2420 (17.1%) |
| Behavioral Syndrome | 2,556 / 17288 (14.7%) | 1,245 / 7517 (16.5%) | 251 / 2807 (8.9%) | 494 / 2996 (16.4%) | 157 / 1560 (10.0%) | 409 / 2408 (16.9%) |
| Functional Deficit | 2,731 / 17264 (15.8%) | 1,156 / 7500 (15.4%) | 420 / 2800 (15.0%) | 523 / 2999 (17.4%) | 282 / 1553 (18.1%) | 350 / 2412 (14.5%) |
| Amputation | 373 / 17162 (2.1%) | 143 / 7463 (1.9%) | 92 / 2785 (3.3%) | 68 / 2961 (2.3%) | 31 / 1561 (1.9%) | 39 / 2392 (1.6%) |
| **Systemic Complications** | 6,146 / 427539 (1.4%) | 1,958 / 135905 (1.4%) | 1,554 / 109371 (1.4%) | 1,286 / 100426 (1.2%) | 530 / 38095 (1.3%) | 818 / 43742 (1.8%) |
| Renal | 3,884 / 6032 (64.3%) | 846 / 1928 (43.8%) | 1,100 / 1515 (72.6%) | 908 / 1269 (71.5%) | 389 / 519 (74.9%) | 641 / 801 (80.0%) |
| Respiratory/Acute Pulmonary Edema | 1,885 / 5922 (31.8%) | 650 / 1911 (34.0%) | 515 / 1485 (34.6%) | 396 / 1241 (31.9%) | 136 / 518 (26.2%) | 188 / 767 (24.5%) |
| Septicemia | 555 / 5815 (9.5%) | 248 / 1901 (13.0%) | 109 / 1424 (7.6%) | 102 / 1227 (8.3%) | 37 / 508 (7.2%) | 59 / 755 (7.8%) |
| Shock | 1,723 / 5877 (29.3%) | 901 / 1912 (47.1%) | 329 / 1459 (22.5%) | 261 / 1233 (21.1%) | 103 / 509 (20.2%) | 129 / 764 (16.8%) |
| **Work related envenomation** | 136,536 / 427078 (31.9%) | 49,228 / 135556 (36.3%) | 30,638 / 108664 (28.2%) | 31,952 / 100306 (31.8%) | 13,119 / 38674 (33.9%) | 11,599 / 43878 (26.4%) |
| **Evolution of the case** |  |  |  |  |  |  |
| Cure | 433,235 / 435474 (99.4%) | 138,853 / 139612 (99.4%) | 111,866 / 112613 (99.3%) | 100,719 / 101083 (99.6%) | 37,805 / 37926 (99.6%) | 43,992 / 44240 (99.4%) |
| Death from envenomation | 2,039 / 435474 (0.4%) | 691 / 139612 (0.4%) | 688 / 112613 (0.6%) | 324 / 101083 (0.3%) | 108 / 37926 (0.2%) | 228 / 44240 (0.5%) |
| Death from other causes | 200 / 435474 (0.0%) | 68 / 139612 (0.0%) | 59 / 112613 (0.0%) | 40 / 101083 (0.0%) | 13 / 37926 (0.0%) | 20 / 44240 (0.0%) |
