# Supplementary material for: Assessing territorial disparities in snakebite surveillance data in Brazil: Implications for public health
Source: PLoS Negl Trop Dis. 2026 Jan 16;20(1):e0013873. doi: 10.1371/journal.pntd.0013873 (PMC12810854; doi:10.1371/journal.pntd.0013873)
Supplement: S2 Table — Completeness percentages for key sociodemographic, clinical, and treatment-related variables reported in SINAN, stratified by Brazilian geographic region. Classification of completeness levels follows Romero and Cunha’s criteria: excellent (≥95%), good (90–94.9%), regular (80–89.9%), poor (50–79.9%), and very poor (<50%). (DOCX) [file pntd.0013873.s002.docx]

**Supplementary Table 2**

|  | **Total**  **Complete (%)** | **North**  **Complete (%)** | **Northeast**  **Complete (%)** | **Southeast**  **Complete (%)** | **South**  **Complete (%)** | **Midwest**  **Complete (%)** |
| --- | --- | --- | --- | --- | --- | --- |
| Region | 55.08 - Poor | 47.50 - Very poor | 56.73 - Poor | 56.92 - Poor | 67.59 - Poor | 60.19 - Poor |
| Biological sex | 99.98- - Excellent | 99.99- Excellent | 99.98- - Excellent | 99.98- Excellent | 99.99- - Excellent | 99.98- Excellent |
| Pregnant | 97.35- Excellent | 98.40- Excellent | 96.05- Excellent | 96.96- Excellent | 98.52- - Excellent | 97.38- Excellent |
| Ethnicity | 91.51 - Good | 95.69- - Excellent | 86.9 - Average | 89.44 - Average | 96.21- - Excellent | 91.36 - Good |
| Education | 60.40 - Poor | 66.87 - Poor | 52.9 - Poor | 55.97 - Poor | 70.59 - Poor | 61.54 - Poor |
| State of residence | 99.85- - Excellent | 99.63- - Excellent | 100- - Excellent | 99.99- Excellent | 99.85- - Excellent | 99.83- Excellent |
| Resident Municipality | 99.85- - Excellent | 99.63- - Excellent | 100- - Excellent | 99.99- Excellent | 99.85- - Excellent | 99.83- Excellent |
| Occupation | 52.64 - Poor | 54.17 - Poor | 49.31 - Very poor | 52.3 - Poor | 60.37 - Poor | 51.04 - Poor |
| Elapsed Time Sting/Attendance | 93.45 - Good | 94.41 - Good | 90.97 - Good | 93.74 - Good | 95.64- - Excellent | 94.6 - Good |
| Location of the bite | 98.27- - Excellent | 99.13- - Excellent | 96.59- - Excellent | 98.62- - Excellent | 98.9- - Excellent | 98.69- Excellent |
| Local Manifestations | 97.87- Excellent | 98.21- Excellent | 96.63- - Excellent | 98.5- Excellent | 98.57- Excellent | 98.1- Excellent |
| Ache | 91.54 - Good | 94.26 - Good | 87.74 - Average | 92.06 - Good | 92.51 - Good | 91 - Good |
| Edema | 91.10 - Good | 94.09 - Good | 86.82 - Average | 91.66 - Good | 92.43 - Good | 90.56 - Good |
| Ecchymosis | 89.82 - Average | 93.03 - Good | 85.16 - Average | 90.41 - Good | 91.86 - Good | 88.99 - Average |
| Necrosis | 89.60 - Average | 92.89 - Good | 84.9 - Average | 90.1 - Good | 91.69 - Good | 88.71 - Average |
| Other locations | 87.76 - Average | 91.05 - Good | 83.42 - Average | 88.32 - Average | 89.7 - Average | 85.94 - Average |
| Other locations(specify) | 6.94 - Very poor | 3.56 - Very poor | 8.79 - Very poor | 7.77 - Very poor | 10.59 - Very poor | 7.89 - Very poor |
| Systemic manifestations | 93.99 - Good | 94.49 - Good | 91.74 - Good | 95.06- Excellent | 96.01- - Excellent | 94.4 - Good |
| Neuroparalytic | 16.95 - Very poor | 17.10 - Very poor | 18.89 - Very poor | 15.54 - Very poor | 13.39 - Very poor | 17.61 - Very poor |
| Hemorrhagic | 16.90 - Very poor | 17.11 - Very poor | 18.77 - Very poor | 15.5 - Very poor | 13.38 - Very poor | 17.53 - Very poor |
| Specify vagal (vomiting/diarrhea) | 16.92 - Very poor | 17.09 - Very poor | 18.76 - Very poor | 15.49 - Very poor | 13.4 - Very poor | 17.76 - Very poor |
| Myolytic / hemolytic | 16.81 - Very poor | 17.01 - Very poor | 18.61 - Very poor | 15.43 - Very poor | 13.35 - Very poor | 17.47 - Very poor |
| kidney (oliguria/anuria) | 16.77 - Very poor | 17.00 - Very poor | 18.55 - Very poor | 15.38 - Very poor | 13.33 - Very poor | 17.44 - Very poor |
| Other systemic | 16.54 - Very poor | 16.65 - Very poor | 18.44 - Very poor | 15.22 - Very poor | 13.16 - Very poor | 17.08 - Very poor |
| Other systemic (specify) | 3.71 - Very poor | 2.52 - Very poor | 5.02 - Very poor | 3.65 - Very poor | 3.65 - Very poor | 4.28 - Very poor |
| Clotting time | 44.38 - Very poor | 44.86 - Very poor | 40.1 - Very poor | 44.4 - Very poor | 51.55 - Poor | 48.4 - Very poor |
| Case Classification | 94.00 - Good | 95.15- - Excellent | 91.21 - Good | 94.85 - Good | 95.86- - Excellent | 94.45 - Good |
| Serum therapy | 95.96- - Excellent | 97.90- - Excellent | 93.19 - Good | 95.7- - Excellent | 96.92- - Excellent | 97.08- Excellent |
| Local complications | 86.62 - Average | 86.25 - Average | 82.53 - Average | 89.28 - Average | 91.31 - Good | 88.88 - Average |
| Secondary Infection | 4.08 - Very poor | 5.53 - Very poor | 2.62 - Very poor | 3.05 - Very poor | 4.15 - Very poor | 5.52 - Very poor |
| Extensive Necrosis | 3.98 - Very poor | 5.43 - Very poor | 2.52 - Very poor | 2.97 - Very poor | 4.08 - Very poor | 5.41 - Very poor |
| Behavioral Syndrome | 3.96 - Very poor | 5.42 - Very poor | 2.49 - Very poor | 2.94 - Very poor | 4.06 - Very poor | 5.38 - Very poor |
| Functional Deficit | 3.96 - Very poor | 5.41 - Very poor | 2.49 - Very poor | 2.95 - Very poor | 4.04 - Very poor | 5.39 - Very poor |
| Amputation | 3.93 - Very poor | 5.38 - Very poor | 2.47 - Very poor | 2.91 - Very poor | 4.06 - Very poor | 5.35 - Very poor |
| Systemic Complications | 97.99- Excellent | 97.94- Excellent | 97.16- Excellent | 98.67- Excellent | 99.03- - Excellent | 97.76- Excellent |
| Renal | 1.38 - Very poor | 1.39 - Very poor | 1.35 - Very poor | 1.25 - Very poor | 1.35 - Very poor | 1.79 - Very poor |
| Respiratory/Acute Pulmonary Edema | 1.36 - Very poor | 1.38 - Very poor | 1.32 - Very poor | 1.22 - Very poor | 1.35 - Very poor | 1.71 - Very poor |
| Septicemia | 1.33 - Very poor | 1.37 - Very poor | 1.27 - Very poor | 1.21 - Very poor | 1.32 - Very poor | 1.69 - Very poor |
| Shock | 1.35 - Very poor | 1.38 - Very poor | 1.3 - Very poor | 1.21 - Very poor | 1.32 - Very poor | 1.71 - Very poor |
| Work related envenomation | 84.78 - Average | 84.26 - Average | 79.67 - Poor | 87.99 - Average | 91.8 - Good | 87.16 - Average |
| Evolution of the case | 86.45 - Average | 86.78 - Average | 82.57 - Average | 88.67 - Average | 90.02 - Good | 87.88 - Average |
| Date of Death | 73.38 - Poor | 24.83 - Very poor | 22.55 - Very poor | 12.59 - Very poor | 5.18 - Very poor | 8.22 - Very poor |
| Closing date | 95.63- - Excellent | 94.90 - Good | 94.17 - Good | 97.26- Excellent | 98.07- Excellent | 96.22- Excellent |
| Typing date | 58.34 - Poor | 58.47 - Poor | 54.61 - Poor | 59.34 - Poor | 65.13 - Poor | 60.03 - Poor |
