# Supplementary figures and images for: Assessing territorial disparities in snakebite surveillance data in Brazil: Implications for public health
Source: PLoS Negl Trop Dis. 2026 Jan 16;20(1):e0013873. doi: 10.1371/journal.pntd.0013873 (PMC12810854; doi:10.1371/journal.pntd.0013873)

**Supplementary Figure 1**


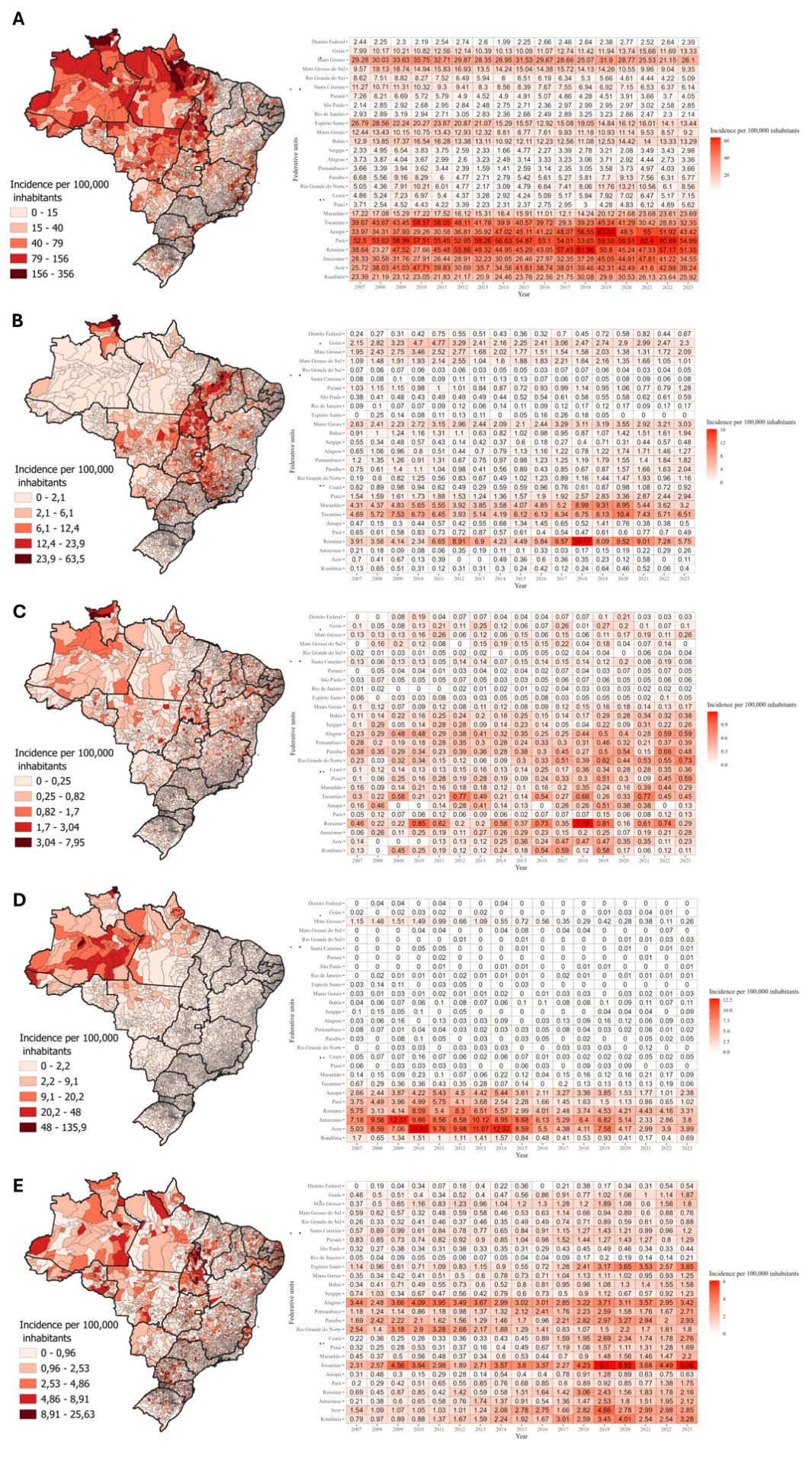

Supplement: S1 Fig — Spatial distribution of snakebite envenoming notifications across Brazilian municipalities based on SINAN data from 2007 to 2023. Higher case densities are observed in the North region, particularly in Amazonian states. Municipalities with no registered cases are shown in white. A – Bothrops; B – Crotalus; C – Micrurus; D – Lachesis; E – Non-venomous snakes. In addition, a heatmap illustrates the temporal trends of snakebite notifications across Brazilian states during the study period. Source of shapefiles: Brazilian Institute of Geography and Statistics (IBGE), 2023. Shapefiles are available under the Creative Commons Attribution 4.0 License (CC BY 4.0): https://www.ibge.gov.br/geociencias/organizacao-do-territorio/malhas-territoriais/15774-malhas.html?=&t=acesso-ao-produto. (DOCX) [file pntd.0013873.s003.docx]
